# Surgery nurses' telephone communication: a mixed methods study with a special focus on newcomers' calls

**DOI:** 10.1002/nop2.128

**Published:** 2018-02-25

**Authors:** Esther González‐Martínez, Katarzyna Piotrowska, Anca‐Cristina Sterie, Carla Vaucher

**Affiliations:** ^1^ Department of Social Sciences University of Fribourg Fribourg Switzerland; ^2^ Present address: Geriatric Palliative Medicine Lausanne University Hospital Lausanne Switzerland

**Keywords:** conversation analysis‐based coding, descriptive statistics, hospital, internal communication, intra‐ and interprofessional communication, mixed methods, newcomer nurses, nurses, requests, surgery department, telephone calls

## Abstract

**Aims:**

The aim of this study was (i) to document the main features of surgery nurses' telephone calls, with a special focus on newcomers' calls; and (ii) to identify the main activities accomplished during the newcomers' calls.

**Design:**

Mixed methods study.

**Methods:**

We audio recorded telephone calls internal to the hospital in two surgery nursing stations. We performed statistical descriptive analysis of the total collection of calls and of those specifically involving the newcomers and compared both sets. We also performed conversation analysis‐based coding of the main activities accomplished during newcomers' calls.

**Results:**

Surgery nurses' telephone calls are extremely brief, predominantly nurse initiated and take place with a wide range of interlocutors who, for the most part, use mobile phones. The newcomers' calls are only slightly longer, take place with a more limited, but still wide, range of interlocutors and are even more often nurse initiated. The main activities of newcomers' calls are requests and activities related to connecting relevant interlocutors.

## 1 INTRODUCTION

Hospital nurses play an increasingly substantial and acknowledged part in care coordination that requires frequent communication with many different co‐workers (Allen, 2014; Apker, 2012). Healthcare professionals, including nurses, preferably rely on synchronous communication forms, mainly face‐to‐face and telephone interaction, when contacting hospital colleagues (Coiera, Jayasuriya, Hardy, Bannan, & Thorpe, 2002; Woloshynowych, Davis, Brown, & Vincent, 2007; Wu et al., 2010). Professional telephone use is bound to grow in importance in hospitals worldwide as the number, spatial scattering and interdependence of care providers increase, work pace accelerates and devices offer new options for enhanced verbal communication (Wu et al., 2012). Concomitantly, nurses and nursing students are required to adopt improved communication procedures for telephone referrals (Cunningham et al., 2012; Haig, Sutton, & Whittington, 2006; on SBAR), verbal telephone orders (Wakefield et al., 2012) and reports of critical information (Barenfanger et al., 2004, on Read‐Back). However, we still know fairly little about the prevalence and main characteristics of nurses' calls with hospital co‐workers and even less about newcomers' calls as they happen in real‐life situations. For this study, we audio recorded and analysed telephone calls, internal to the hospital, in two surgery nursing stations of an acute‐care facility in Switzerland.

### 1.1 Background

Studies on verbal telephone communication at the hospital have mostly concentrated on calls between healthcare professionals and patients. A few studies have focused on calls between hospital coworkers, investigating for instance the telephone communication of physicians (Aziz et al., 2005; Ortega, Taksali, Smart, & Baumgaertner, 2009; Soto, Chu, Goldman, Rampil, & Ruskin, 2006), among the staff of general internal medicine units (Lo, Wu, Morra, Lee, & Reeves, 2012; Whitlow, Drake, Tullmann, Hoke, & Barth, 2014; Wu et al., 2010), between floor nurses and on‐call physicians (Bernstam et al., 2007) and between nurses in medical emergency centres and physicians on duty (Tjora, 2000). The majority of the existing studies revolve around safety issues related to mobile phones (Myerson & Mitchell, 2003) and their pros and cons compared with pager‐mediated contact (Lo et al., 2012; Ortega et al., 2009; Soto et al., 2006) and landline telephone use (Hanada, Fujiki, Nakakuni, & Sullivan, 2006). They also examine the usefulness of telephone communication between physicians in terms of continuity of care (Blankenship, Menapace, Fox, & Frey, 1999; Crone, 1987), ways remote and technology‐mediated work communication may balance the nurse–physician professional relationship (Tjora, 2000) and the reasons for after‐hours nurse‐to‐physician calls (Bernstam et al., 2007). Previous studies also highlight the importance of shared understanding among co‐workers of telephone conversation terms of use to prevent interruptions (Solvoll, Scholl, & Hartvigsen, 2013), unnecessary calls (Bernstam et al., 2007; Wu et al., 2010) and communication errors (Rabøl et al., 2011).

Telephone communication is an important part of nurses' work and is closely connected to care organization. Nevertheless, research has repeatedly shown that new graduate nurses experience challenges when communicating with co‐workers, handling organizational discussions with other hospital personnel and answering the telephone (Maben, Latter, & Macleod Clark, 2006). Wangensteen, Johansson, and Nordström (2008) note that novice nurses often lack the competences required to work as members of an interdisciplinary team, not knowing, for instance, whether they should call the physician or not. Fink, Krugman, Casey, and Goode (2008) report the need for novice nurses to work on assertiveness with physicians and to learn to give orders to assistant personnel with confidence. O'Shea and Kelly (2007) identified several tasks as problematic for novice nurses: organizing investigations and referrals, contacting specialized clinical nurses and other healthcare providers such as dieticians and physiotherapists and booking tests and ordering items from the pharmacy (pp. 1538 & 1540). In response to these issues, nursing training programmes now incorporate teaching on interprofessional communication (Chant, Jenkinson, Randle, & Russell, 2002), sometimes focusing specifically on telephone transactions (Krautscheid, 2008).

Most of the above‐mentioned studies are based on simulated, experimental or intervention‐driven communication situations and/or used questionnaires, interviews and in situ observations as data collection techniques. There is a lack of naturalistic studies and basic information like the identity of the nurses' most common interlocutors, the number, length and direction of the calls and the purposes they serve. The present study addresses nurses' telephone communication, as practiced in situ and in real time during recorded real‐life hospital calls. The study specifically aims to: (i) document the main features of surgery nurses' telephone calls with hospital personnel, including a special focus on newcomer nurses' calls; (ii) identify the main activities accomplished during the newcomers' calls.

## 2 THE STUDY

### 2.1 Design

The study was part of a larger research project on hospital personnel telephone communication adopting the interactional workplace studies framework (Arminen, 2005) and based on ethnographic fieldwork and systematic audio recording of calls. For this particular mixed methods study, we combined statistical descriptive analysis and conversation analysis‐based coding of calls recorded in two surgery nursing stations.

### 2.2 Setting

Our study was conducted in the surgery department of an acute‐care hospital with approximately 200 beds in Switzerland. The surgery department comprised four different care units, two stationary and two ambulatory, each of them with its own nursing station. The study concentrated on telephone calls made or answered using the landline phones of the two stationary units, which we called units one and two. Both units provided similar care services related to general, orthopaedic, trauma, vascular and visceral surgery. Each care unit had 15 double rooms. The nursing staff of each unit was composed of approximately 35 people (1 head nurse, 24–25 nurses, 4–6 aides, 2–3 nursing interns and nursing students). In both units, the landline telephone was used by all categories of nursing personnel and for all kinds of purposes. In both units, mobile phones were used by some of the nurses (for instance, the head nurse, the nurse in charge for the day and the nurses of the night shift), but the activity of these devices was not considered for the study.

### 2.3 Methods

We used a service of the hospital's telephone provider to collect (directly from the hospital switchboard centre) audio recorded calls between the landline telephones of the surgery department's care units one and two and 155 telephone numbers in the hospital. These telephone numbers corresponded to 111 mobile and 44 landline phones. They were attributed to 123 different interlocutors, one single interlocutor sometimes having two different numbers, one landline and one mobile. An interlocutor could designate an individual person, a physician for instance, or a collective entity, such as the laboratory. The interlocutors belonged to 19 hospital departments.

The 24/7 recording service automatically recorded calls from the time the caller finished dialling the number to the time the handset of the nursing station landline phone was hung up. Calls were collected this way over a period of 174 consecutive days. Based on the information provided automatically by the recording system, a researcher (Author 1) created an Excel^®^ file with 9,931 entries, each corresponding to a different recorded call; we named the resulting collection the NTH set. It includes information on the date, time, length and direction (incoming or outgoing) of the call, the caller's and the called party's telephone numbers and the devices used (landline or mobile phone).

All the calls were listened to twice, each time by a different member of the research team (Author 3, Author 4), to identify the ones made or answered by the three newly graduated nurses in their first year of employment who agreed to participate in the study. Two female newcomer nurses were working in unit one and the other, a male, was in unit two. This collection of 374 calls forms the NTH‐3 subset.

### 2.4 Analysis

We analyse the NTH database, which includes the NTH‐3 subset, with descriptive statistics (number of events, percentages, mean, median and standard deviation) using SPSS^®^ software. We examined the nurses' interlocutors and the devices they used, whether landline or mobile phone, as well as the day of the week and the time, direction and length of the calls and compared the calls of the general nursing staff with those of the newcomers. The aim was merely to describe the telephone activity of the general nursing staff and the three newcomer nurses during the 6 months of the study. We did not infer that the identity of the group—the fact of being newcomers specifically—was in itself the reason for any identified differences between the two sets, which in any case did not refer to two distinct homogenous populations.

We also fully transcribed the newcomers' calls—the NTH‐3 subset containing 374 calls—applying the conversation analysis conventions (Jefferson, 2004). We then coded the calls manually according to the interlocutors' main activities (Robinson, 2007; Stivers, 2015), which resulted in numerical results. The coding of the transcripts followed the orientations of the call interlocutors, who exhibited their own understanding of the ongoing activities (Koenig & Robinson, 2014).

The research team's previous scientific publications (cf. Sterie, 2015; Sterie & González‐Martínez, 2017) presented qualitative conversation analysis of specific interactional phenomena found in a few selected newcomers' calls. In this article, for the first time, we present the quantitative results of the statistical descriptive analysis and the conversation analysis‐based coding in their entirety.

Several steps were taken to ensure the reliability of the audio recordings (Peräkylä, 2004); we discussed the details of the recording procedure with the staff in advance to secure their support; the automatic recording system provided for minimal interference in work routines and the recordings were made uninterruptedly over an extended period of 6 months, thus becoming a common feature of the setting. The descriptive statistical analysis was done by one researcher (Author 2) and reviewed by another (Author 1). The transcription of the recordings was done collaboratively by two researchers (Author 3 and Author 4). The coding was done independently by two researchers (Author 1 and Author 3). Discrepancies between transcribers and coders were subsequently examined during team data sessions until agreement was reached.

### 2.5 Ethics

The research protocol was approved by the hospital's board of directors, the body responsible for ethical reviews and included ethical requirements specifically related to the collection of recordings in a clinical setting (Broyles, Tate, & Happ, 2008). We collected only internal calls between a predetermined set of telephone numbers. All the employees involved in the study were informed of the research project and the fact that recordings would be made. They were given the opportunity to freely opt out of the study if they wished to. We kept only the calls of interlocutors who agreed to be recorded. Moreover, a pre‐recorded voice message reminded callers that the calls were being recorded for research purposes. We anonymized all personal information related to the telephone interlocutors and any persons referred to during the calls. According to the research protocol, we transcribed only the calls made or answered by the three newcomers, who gave voluntary written informed consent, for further conversation analysis.

## 3 RESULTS

### 3.1 The main features of the calls

#### 3.1.1 Number of calls and interlocutors

The NTH set comprises 9,931 calls made or answered by the general nursing staff of surgery care units one and two over a period of 174 consecutive days. Table 1 shows that the staff had calls with all the departments (*N* = 19) participating in the study. The calls involved 129 telephone numbers matching 109 interlocutors of the 123 set to be recorded, which attests to the fact that the nursing staff was in contact with a wide range of hospital personnel.

**Table 1:** Features of calls to/from surgery nursing stations (NTH set)

Interlocutors Department (interlocutors)^a^		No. of calls *n*	Phone numbers		Direction		Device		Duration (mm:ss.00)		
			Recorded set	Utilized	Outgoing	Incoming	Mobile	Landline	Mean	*SD*	Median
1	Surgery (care unit head nurses, nursing stations, physicians, secretaries)	3,065	70	59	2,707	358	2,795	270	00:45.61	00:40.80	00:36.00
2	Transportation services (department head, porters—main number)	1,068	3	3	1,054	14	1,064	4	00:28.25	00:21.05	00:22.00
3	Recovery room (main numbers, anaesthesia nurse manager)	1,020	4	3	312	708	103	917	00:36.20	00:24.73	00:28.00
4	Reception (main numbers)	850	2	2	726	124	0	850	01:41.15	01:35.01	01:10.00
5	Nutrition services (dieticians—main numbers, meal coordinator dieticians)	594	3	3	432	162	397	197	01:07.93	00:54.18	00:55.00
6	Intensive Care (department head nurse, desks)	554	4	4	200	354	2	552	00:50.01	00:37.23	00:33.50
7	Social Services (*department director*, deputy department director, social workers' office, social workers, liaison nurses)	525	16	13	422	103	525	0	01:02.58	01:23.97	00:34.00
8	Laboratory (main number)	467	1	1	443	24	0	467	00:43.02	00:41.96	00:35.00
9	Kitchen (main number, room service operators—main number)	396	2	2	387	9	354	42	00:38.08	00:21.83	00:32.00
10	Emergency (department head nurse, *deputy department head nurse*, triage nurse managers)	353	6	5	236	117	224	129	00:57.82	00:44.06	00:46.00
11	Pharmacy (main numbers)	259	2	2	251	8	92	167	01:11.81	01:11.31	00:55.00
12	Physiotherapy (department head, deputy department head, physiotherapists)	210	20	17	204	6	210	0	00:52.25	00:42.53	00:46.00
13	Medical imaging (administrative office—main number)	182	1	1	134	48	0	182	00:47.95	00:51.55	00:33.00
14	Nursing directorate (branch nurse manager, surgery department head nurse)	139	3	3	44	95	138	1	01:09.35	01:01.80	00:48.00
15	Bed Management (*department head*, secretaries, surgical scheduling office)	71	11	4	51	20	27	44	00:58.11	00:52.43	00:47.00
16	Occupational therapy (hospital treatment—main numbers, ambulatory treatment—main numbers)	12	4	4	11	1	9	3	01:01.58	00:38.47	01:03.50
17	Security (hotline)	12	1	1	10	2	0	12	00:54.92	00:20.98	00:56.50
18	Technical service (hotline)	11	1	1	11	0	0	11	00:36.55	00:20.03	00:39.00
19	Outpatient clinic (administrative office—main number)	7	1	1	1	6	7	0	00:47.43	00:32.28	00:27.00
Unidentified		136	b	b	82	54	b	b	00:48.79	00:56.43	00:33.00
Total		9,931^c^	155	129	7,718	2,213	5,947	3,848	00:51.37	00:53.74	00:35.00

a Interlocutors (*n* = 123), distributed by department (*n* = 19), whose numbers were set to be recorded. In italics: interlocutors (*n* = 3) for which no recorded calls were collected.

b non‐relevant/non‐available information for calls with unidentified interlocutors.

c 9,795 calls with identified interlocutors out of 9,931 recorded calls.

The NTH‐3 subset comprises 374 calls made or answered by three newly graduated nurses who were in their first year of employment in the surgery department. Table 2 shows that the newcomers were in contact with fewer hospital personnel (49 interlocutors using 56 telephone numbers) than the nursing staff as a whole. Yet, they still communicated with a significant number of hospital departments: 14 of 19.

**Table 2:** Features of calls made/answered by newcomer surgery nurses (NTH‐3 subset)

Interlocutors Department (interlocutors)^a^		No. of calls *n*	Phone numbers	Direction		Device		Duration (mm:ss.00)		
			Utilized	Outgoing	Incoming	Mobile	Landline	Mean	*SD*	Median
1	Surgery (care unit head nurses, nursing stations, physicians, secretaries)	148	30	131	17	134	14	00:50.08	00:36.03	00:41.50
2	Transportation services (department head, porters—main number)	54	2	53	1	54	0	00:27.09	00:28.93	00:20.00
3	Recovery room (main numbers)	41	2	22	19	7	34	00:43.98	00:26.39	00:34.00
4	Reception (main numbers)	27	2	25	2	0	27	02:26.67	01:40.24	02:07.00
5	Laboratory (main number)	24	1	22	2	0	24	00:39.46	00:18.88	00:35.50
6	Nutrition services (dieticians—main number, meal coordinator dieticians)	19	3	12	7	8	11	01:27.74	00:55.45	01:03.00
7	Emergency (department head nurse, triage nurse managers)	18	4	11	7	11	7	01:00.11	00:23.53	00:55.00
8	Social services (social workers, liaison nurses)	16	4	13	3	16	0	01:26.50	00:49.46	01:09.00
9	Intensive care (desks)	9	2	5	4	0	9	00:51.67	00:23.18	00:48.00
10	Pharmacy (main numbers)	7	2	7	0	2	5	01:20.00	00:50.28	01:01.00
11	Kitchen (room service operators—main number)	6	1	6	0	6	0	00:40.33	00:27.20	00:29.00
12	Medical imaging (administrative office—main number)	3	1	3	0	0	3	00:37.33	00:11.15	00:35.00
13	Bed management (surgical scheduling office)	1	1	0	1	0	1	01:04.00	00:00.00	01:04.00
14	Physiotherapy (physiotherapist)	1	1	1	0	1	0	00:23.00	00:00.00	00:23.00
Total		374	56	311	63	239	135	00:56.64	00:51.90	00:41.00

a Interlocutors (*n* = 49), distributed by department (*n* = 14), for which recorded calls were collected.

#### 3.1.2 Types of interlocutors

The surgery nursing stations' most common interlocutors were their own department (30.86% of all calls) and transportation services (10.75%) (Table 1).^1^ The physicians working in the surgery department were the individuals with whom the nursing stations were most in contact (25.79% of all calls). Among this category of interlocutors, residents (*médecins assistants*) were the ones that the nurses talked to most often (71.03% of calls with physicians) (Table 3).

**Table 3:** Distribution of calls among physicians (NTH set and NTH‐3 subset)

Interlocutors (*rank in French*)^a^	NTH		NTH‐3	
	No. of calls	%	No. of calls	%
Head of department (*médecin chef du département*)	29	1.13	2	1.54
Head physicians (*médecins chefs*)	57	2.23	3	2.31
Associate physicians (*médecins adjoints*)	80	3.12	2	1.54
Affiliate physicians (m*édecins spécialistes*)	13	0.51	0	0.00
Attending physicians (*chefs de clinique)*	325	12.69	13	10.00
Fellows (*chefs de clinique adjoints*)	238	9.29	10	7.69
Residents (*médecins assistants*)	1,819	71.03	100	76.92
Total	2,561	100.00	130	100.00

a Listed from highest to lowest rank according to the Swiss teaching hospital system.

Table 2 shows that the newcomers' most common interlocutors were the same, but in higher proportions, the surgery department (39.57%) and transportation services (14.44%). The proportion is also higher when it comes to calls between the newcomers and physicians (34.76%) and, among them, residents (76.92% of calls to physicians) (Table 3).

#### 3.1.3 Direction of the calls

Of the NTH, 77.72% calls were outgoing, namely nurse initiated. However, the general nursing staff answered more calls that it made itself when dealing with a few departments (Table 1). The fact that most calls were nurse initiated was particularly the case for the newcomers, whose calls were 83.16% outgoing. The newcomers made more calls than they answered irrespective of the interlocutors' department (Table 2).

#### 3.1.4 Type of device with regard to interlocutor

The nurses used the station's landline telephone, but most of their interlocutors used a mobile phone when communicating with them (60.71% of all calls with identified interlocutors). In some departments, landline and mobile phone numbers were set to be recorded; but in the end, the vast majority of calls, sometimes all of them, took place between the nursing station landline and a mobile phone. This was the case for calls with the nursing directorate (99.28%), transportation services (99.63%) and social services (100%). In contrast, calls with the intensive care department took place overwhelmingly (99.64%) between a nursing station landline number and one of the department landline numbers, although department mobile phone numbers were also set to be recorded (Table 1). Table 2 shows that the proportion of calls with parties using mobile phones was even higher for the newcomers. Indeed, 63.90% of the calls in the NTH‐3 subset took place between a nursing station landline telephone and a mobile telephone.

#### 3.1.5 Duration of the calls with regard to interlocutor and device

Table 1 shows that the mean duration of the calls was 51.37 s, counting from the end of the dialling of the number, to the time the handset of the nursing station landline phone was hung up.^2^ The longest calls took place with the pharmacy and the briefest calls were with porter services. On average, calls with interlocutors using landline phones lasted 12.64 s longer than calls with interlocutors using mobile phones.

Table 2 shows that the calls with the newcomers were only slightly longer (*M* = 00:56.64) than the calls with the nursing staff. Much like with the NTH calls, the length of the newcomers' calls varied depending on the interlocutor. The newcomers' calls with interlocutors using a landline phone also lasted longer, 22.52 s, than those with interlocutors using a mobile phone.

Moreover, the variability in the length of the calls, of both the general nursing staff and the newcomers, was higher with interlocutors using a landline phone and also for calls with some specific departments.

#### 3.1.6 Day and time of the calls

The number of calls of the general nursing staff (NTH set) varied depending on the day. Calls on a weekday (mean = 69.65) were, respectively, 1.83 and 2.44 times more numerous than on a weekend day or a bank holiday. The day of the week with the highest number of calls was Friday (mean = 77.57). The recording system operated around the clock, but 97.86% of the recorded calls took place between 7 a.m. and 9 p.m. This is consistent with the system collecting only the calls of the nursing station landline phones and nurses tending to switch to mobile phones at night. The hourly distribution of the calls was very similar on weekdays, showing two periods of the day when the calls were particularly numerous: after the morning medical round and the care routines were performed (11.67% of the calls) and following the afternoon nursing handover and before the doctors' visits (10.60% of the calls).

The newcomers' calls had very similar features: calls on a weekday (mean = 2.56) were, respectively, 2.34 and 1.71 times more numerous than on a weekend day or a bank holiday. 96.45% of the recorded calls took place between 7 a.m. and 9 p.m., and the hourly distribution of the calls was very similar on weekdays and weekend days.

### 3.2 Main activities in the newcomers' calls

In most of the newcomers' calls (*N* = 289 out of 374), the dialling resulted in a single telephone connection where one single conversation with one single main activity took place. Interlocutors thus produced monofocal conversations (Wakin & Zimmerman, 1999) where they rapidly transacted a single business. However, some calls were initially made to one telephone number and then transferred to another number, resulting in two telephone connections. During a single connection, two distinct conversations sometimes took place, each of them corresponding to a spate of talk between different interlocutors. Moreover, some conversations had no main activity, for instance when the called person immediately stated that he or she was not available to talk. In contrast, a few conversations had more than one main activity, for instance if a nurse called her manager with two matters on the agenda: she first addressed a request for instruction, the two parties discussed it and then she moved on to produce an unrelated report on a patient. This diversity of organizations accounted for a total of 356 instances of main activities out of 374 calls (Table 4).

**Table 4:** Main activities, with interlocutors, in calls made/answered by newcomer surgery nurses (NTH‐3 subset)

Main activity	No. of instances		No. of instances by interlocutor						
	*N*	%^a^	Surgery Physician	Transport Services	Reception	Recovery Room	Laboratory	Physician Secretary	Other
None: connecting	39	b	26	1	0	2	3	1	6
Requesting									
Request about medical treatment	108	30.34	87	0	0	4	0	0	17
Request about patient transfer	74	20.79	0	47	0	20	0	0	7
Request about object transfer	35	9.83	1	5	0	3	3	0	23
Request for contact	35	9.83	3	1	24	1	0	1	5
Request about patient discharge	29	8.15	15	0	0	0	0	0	14
Request about laboratory tests	16	4.49	0	0	0	0	16	0	0
Request about appointments	12	3.37	0	0	0	0	0	9	3
Request for information for relatives	8	2.25	0	0	1	7	0	0	0
Request about patient entry	6	1.69	0	0	0	0	0	0	6
Request about other matters	14	3.93	5	0	1	0	0	0	8
Transferring information	15	4.21	5	0	0	3	2	0	5
Transferring a call	3	0.84	0	0	2	1	0	0	0
Giving an order	1	0.28	1	0	0	0	0	0	0
Total main activities	356	100.00	117	53	28	39	21	10	88

a percentage of total of main activities.

b non‐relevant information for conversations without a main activity.

The coding of the main activities showed that requests, where one speaker asked his or her interlocutor, more or less overtly, to do something, were by far the most frequent main activity accomplished over the phone (*N* = 337 of 356 identified main activities; 94.67%). In contrast to directives, requests display at least some minimal orientation to the recipient having a say in the future conduct (Craven & Potter, 2010). In 85.76% of the requests, the newcomer was the requester and his or her interlocutor the requestee. Excerpt 1 provides an example of a request.^3^ A newcomer nurse (Amaryse/May) calls the main number of transportation services. A porter on duty (Ronaldo/Ron) picks up the phone (1).
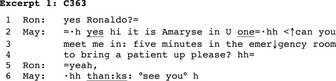


Following self‐identification, Amaryse addresses a request for transportation to Ronaldo (2–4). She displays entitlement to produce the request but orients towards the porter having a say about his future conduct using the interrogative format “can you.” Moreover, she frames her utterance as a request with a turn‐final “please” token. Ronaldo commits to granting the request; Amaryse thanks him and immediately moves to closure. Sterie (2015) and Sterie and González‐Martínez (2017) present additional request formats used by the newcomers.

A total of 108 requests were about medical treatments, related to adding/changing patients' medicine, changing their diet, inspecting and redoing surgical wound dressings and requesting a physician's intervention for problematic medical situations. 80.56% of these requests were produced while the newcomer was in contact with a surgery physician.

A total of 109 requests were about transfer issues, whether of patients (*N* = 74) or objects (*N* = 35) such as medical tools, drugs and food. The most frequent interlocutors for patient transfer requests were transportation services (63.51%) and the recovery room (27.03%) since the nurses needed to go there and take the patients back to their own rooms.

Requests for contact (*N* = 35) were oriented towards asking the call taker to get in contact with or to facilitate getting in contact with a third party. 68.57% of such requests were produced by the newcomers calling the hospital reception, but many also involved the nurse answering incoming calls from interlocutors trying to get in touch with hospital staff or patients through the nursing station.

Most of the requests about patient discharges (*N* = 29) concerned preparing the discharge papers and organizing home transportation and home‐care services. The newcomers produced this activity with physicians (51.72% of such requests) and, in a lesser proportion, with other hospital staff like social workers and dieticians.

Requests related to laboratory tests (*N* = 16) were made either by the newcomers to the laboratory or the other way around, for instance when the laboratory called about a missing blood tube for a previously ordered test. Regular orders were usually placed using paper forms. Telephone communication was used mostly in the event of unusual, urgent or out‐of‐hours requests or when nurses needed to amend or cancel a previous order.

Requests about appointments (*N* = 12) involved the nurses calling mainly physicians' secretaries. Requests for information for relatives (*N* = 8) happened mainly when the nurses called the recovery room to obtain news about a patient. The newcomers produced and handled requests about patient entry (*N* = 6) when the emergency department sought to allocate a new patient to the surgery care unit. Other requests (*N* = 14) concerned, among other topics, administrative issues, such as asking for a patient's identification number or card, technical assistance or help from other nurses.

Besides requests, the parties to the calls produced informings (Heritage, 1984) as main activities (*N* = 15). In this case, the business of the conversation was transferring a piece of information. The types of information and the nurses' interlocutors were highly varied. Excerpt 2 reproduces a call whose main activity is transferring information. A newcomer nurse (Amaryse/May) calls the Intensive Care Department; Anaïs (Ana) picks up the phone.
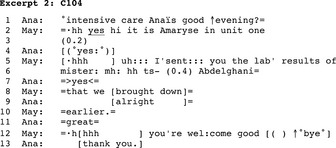


It transpires from the conversation that a patient was previously transferred from surgery to intensive care. Amaryse calls intensive care and informs Anaïs that she has now sent her the patient's laboratory results (5–10). Anaïs shows appreciation and Amaryse responds and moves to closure. The excerpt exemplifies a common use of the phone at the hospital to notify the called person of something sent through the pneumatic tube system or by fax, computer or other means.

The main activity of a few conversations was transferring another call to the call taker (*N* = 3). This happened for instance when the hospital reception contacted the nursing station with a call for a newcomer coming from a third party. In other calls, it was the nurse who acted as a switchboard operator. In Excerpt 3, a newcomer nurse (Leandra/Lea) calls the recovery room main number and Maeva (Mae) answers the phone.
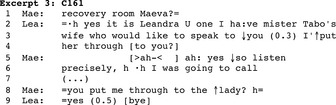


Amaryse displays intent to direct a call from a patient's wife waiting on another line (2–4). Maeva tells Amaryse that she herself was about to call, discusses the patient's return to the care unit (not transcribed) and then resumes with the reason for the call, prompting the nurse to proceed with the transfer. The nurse replies affirmatively and moves to closure.

In only one conversation was the main activity giving an order, which is a directive consisting of telling an individual to do something (Craven & Potter, 2010). In this conversation, Samuel (Sam), a physician, calls the nursing station, a newcomer nurse (Leandra/Lea) replies and the physician enquires whether a patient has already arrived in the unit (non‐transcribed), which is a preliminary to the main activity. In Excerpt 4, following the nurse's answer, the physician enters into the business of the call: giving an order consisting of serving food to the patient on her arrival (4–5).
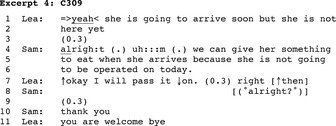


In Excerpt 4, the physician formats his utterance as an informing that nevertheless does more than that he orders the nurse to follow his instructions. Physicians' instructions regarding patients' diets are entered and signed in the medical record and thus have an imperative character. The physician uses informing as a vehicle for his order and provides a reason for serving food but nevertheless shows little orientation towards the nurse having a say about the instructed conduct. On her side, Leandra produces several compliance markers (7) that Samuel may be simply acknowledging in line 8. Then, Leandra responds to Samuel's appreciation token and moves to closure.

In 39 of 380 conversations, the interlocutors did not engage in any proper main activity. During the opening, they realized that the call was no longer appropriate because for instance, the called person was about to reach the nursing station or the caller had failed to reach the intended interlocutor. Once this became known, the interlocutors proceeded to close the call. In Excerpt 5, a newcomer nurse (Amaryse/May) calls a physician on his mobile phone, but it is Franco (Fra), a member of the operating room staff, who answers. 
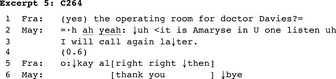


Amaryse receipts Franco's response to the call as conveying that the physician is unavailable and, following self‐identification, tells him that she will call back later (2–3). Franco acknowledges the announced future action and shows acceptance of it; Leandra displays appreciation and moves to closure.

The number of the conversations (*N* = 39) without any proper main activity (inappropriate interlocutors or interlocutors lacking suitable conditions for proceeding with the call), added to the requests for contact (*N* = 35) and activities consisting of transferring a call (*N* = 3), underscores the fact that the newcomers spent a significant part of their time on the phone trying to get in touch with relevant/available interlocutors or putting them in touch with their intended interlocutors.

## 4 DISCUSSION

Reeves et al. (2009) point out that most studies on interprofessional healthcare communication are based on interview data. Nguyen, McElroy, Abecassis, Holl, and Ladner (2015) and Wu et al. (2012) lament the absence of naturalistic studies on clinicians' technology‐mediated communication. Our study examines nurses' telephone practices—a longstanding but understudied activity—based on systematic recordings of real‐life hospital calls. The findings contribute factual detail to the study of hospital nurses' communication practices and have practical implications for nurses' education and work organization.

The hospital is a fast‐paced work environment (Reddy, Dourish, & Pratt, 2006) where nurses operate under significant time pressure (Chan, Jones, & Wong, 2013). We have shown that in this context, nurses are brought to produce extremely brief telephone calls that last on average less than one minute, all interlocutors considered, or less than two minutes when focusing on the interlocutors nurses talk with the longest. Newcomers have very little additional time for their calls compared with the general nursing staff, a fact that reveals the conditions graduates face in their real live work experience. The findings also suggest a higher probability of having a slightly longer call than average when in contact with an interlocutor using a landline phone or when contacting specific interlocutors. Taking contextual factors into consideration and adjusting to them may thus be necessary even when following standardized communication procedures. The literature has abundantly reported on nurses taking a central part in articulation work (Allen, 2014; Apker, 2012; Strauss, 1988). Our findings suggest that this translates into frequent telephone calls where nurses, even newcomers, play an active role as they initiate many more calls than they receive. This also translates into calls to a wide range of hospital personnel. Our study justifies the emphasis that the literature puts on doctor–nurse communication (Tan, Zhou, & Kelly, 2017) proving that physicians are the nurses' most frequent telephone interlocutors. At the same time, it encourages paying close attention to the potential specificities of communication with residents (Weller, Barrow, & Gasquoine, 2011), the category of physicians that nurses are overwhelmingly most in telephone contact with: for instance, reverse distribution of medical expertise in calls between residents and experienced nurses or lack of expertise by young graduates at each end of the line. Our findings also evidence the extent of nurses' interprofessional coordination with non‐medical personnel (Conn et al., 2009; O'Leary, Sehgal, Terrell, & Williams, 2012; Propp et al., 2010; Symon, Long, & Ellis, 1996) as porters are the second most frequent telephone interlocutor of surgery nurses. The fact that nurses may be in contact, with regard to the same patient, with a doctor having the highest levels of medical education, the minute after with a porter lacking any formal training and then contact a social worker to discuss the patient's complex family situation draws our attention to the versatility demanded by telephone communication. For newcomers, learning how to produce concise, yet clear and recipient‐designed requests, is of particular import (Sterie, 2015) since they represent the main activity of most of their calls. Our findings also suggest that a practical implication of nurses performing tasks in a large distributed workspace (Bardram & Bossen, 2005) is that they communicate more often than not with interlocutors who use mobile phones. This invites us to consider ways to adapt to communication plagued with technical disruptions and conducted on the move or at the same time as other activities, when patients' records and tools for decision‐making may be unavailable. Finally, the findings contribute new evidence of the contingency of hospital nurses' communication (González‐Martínez, Bangerter, Lê Van, Navarro, 2016; Reeves et al., 2009). Making and receiving telephone calls in the studied surgery care units was seldom a scheduled activity between specifically targeted interlocutors. Calls are often interstitial activities that non‐intended but available interlocutors need to deal with. The findings thus provide an additional reminder of the real‐life conditions of hospital work that nurses need to be prepared for and adjust to.

Telephone communication requires specific interactional competencies (Jones, 2007; Krautscheid, 2008) to know when, how and whom to call as well as what to say and how to formulate it. It is as difficult to reproduce the conditions of hospital communication at a school as it is problematic to let graduates learn the trade on the job. We advocate for communication training attuned to the realities of hospital work to narrow the theory‐practice gap. While focusing on standardized procedures, information completeness and nurses' assertiveness, instructors should keep in mind that the central communication competence may be context sensitivity.

### 4.1 Limitations

One limitation of the study is that it captures only a part of the telephone activity of the two participating nursing stations. The nurses also used mobile phones and were in contact with additional hospital staff as well as interlocutors outside the hospital and these calls were not recorded. The set of numbers included in the study was nevertheless significant in size and relevant for the nursing staff itself. Another limitation is that the automatically generated data do not make it possible to distinguish between calls, connections and conversations. The system would create a call entry in the database as soon as the caller finished dialling a number regardless of whether, for instance, the call was answered by one interlocutor and then automatically transferred to another number, resulting in two different connections, or answered by one interlocutor and then handed to another co‐present person, resulting in two different conversations. We used the automatically generated information on connected telephone numbers as a basis for attributing interlocutors' identities, except for the coding of the main activities of the newcomers' calls, based on transcripts of the actual conversations. As we did not compare two distinct homogenous populations, we did not identify statistically significant specificities of newcomer calls compared with, for instance, experienced nurses. We merely describe the telephone activity of the general nursing staff, which included a diverse array of personnel and of the three newcomer nurses during the time of the study. Finally, there is a possibility that some interlocutors may have altered their telephone habits because they knew that the calls were being recorded. However, routine telephone practices in a fast‐paced work environment are difficult to alter unilaterally and consistently, for an extended period, in a statistically significant way.

## 5 CONCLUSION

Despite the development of new modes of communication, telephone conversations are and will probably remain a central way for nurses to reach out to hospital co‐workers (Coiera, 2000). The extent and practical import of nurses' telephone activities should be acknowledged so that a full understanding of the manifold character of their work can be achieved. Nurses should be taught, both at school and on the job, how to communicate over the phone in ways suited to the wide range of interlocutors and situations they will encounter. Context‐sensitive systems could be developed to take the location of people or the timing of the communication into consideration, thus reducing calling drawbacks. It is important to ground training measures, communication procedures and technological innovation in research on nurses' day‐to‐day communication practices, based on naturalistic data.

## ACKNOWLEDGMENT

The authors wish to thank the staff of the hospital for their kind participation in the study as well as Elisabeth Lyman for her careful proofreading work.

## CONFLICT OF INTEREST

No conflict of interest has been declared by the authors.

## ENDNOTES

1 In this article, all decimals are rounded to the nearest tenth.

2 The recording system registered the length of calls in minutes and seconds.

3 We have translated the original French conversations into English and replaced the original names of persons and care units with fictional ones. The numbers in parentheses refer to the lines of the excerpt. See the Appendix for a list of the transcription conventions following Jefferson (2004).
